# Supplementary material for: Serum RNAs can predict lung cancer up to 10 years prior to diagnosis
Source: eLife. 2022 Feb 11;11:e71035. doi: 10.7554/eLife.71035 (PMC8884722; doi:10.7554/eLife.71035)
Supplement: Figure 4—source data 1. [file elife-71035-fig4-data1.docx]

| Lung cancer development | No detectable disease | Preneoplasia/Early stage tumor |
| --- | --- | --- |
| Proposed use of RNA biomarkers | **Risk assessment**  Biomarker monitoring of individuals (smokers) with full-time models.  Positive results from any of the models suggest elevated risk of developing lung cancer. | **Early detection**  Biomarker monitoring of elevated risk individuals with prediagnostic models.  Positive results suggest selection for CT monitoring. A positive result might signal preneoplasia or early-stage tumor. Histology specific models can further improve diagnosis accuracy (mean accuracy more than 80%). Bi-yearly monitoring is recommended. |
