# Supplementary material for: Serum RNAs can predict lung cancer up to 10 years prior to diagnosis
Source: eLife. 2022 Feb 11;11:e71035. doi: 10.7554/eLife.71035 (PMC8884722; doi:10.7554/eLife.71035)
Supplement: Supplementary file 1. [file elife-71035-supp1.docx]

|  | **Stage** | | | |  |
| --- | --- | --- | --- | --- | --- |
|  | **Early** (Localized) | **Locally Advanced** (Regional) | **Advanced** (Distant) | **Unknown** | **Controls** |
| **Histology**  NSCLC  SCLC  Others | 0  0  0 | 2  0  0 | 4  0  1 | 0  0  0 | -  -  - |
|  |  |  |  |  |  |
|  |  |  |  |  |  |
| **Sex**  Male  Female | 0  0 | 0  2 | 2  3 | 0  0 | 165  91 |
| **Total Samples** | 0 | 2 | 5 | 0 | 256 |
| **Individuals** |  |  | 260 (nonsmokers) |  |  |
