# Supplementary material for: Serum RNAs can predict lung cancer up to 10 years prior to diagnosis
Source: eLife. 2022 Feb 11;11:e71035. doi: 10.7554/eLife.71035 (PMC8884722; doi:10.7554/eLife.71035)
Supplement: Supplementary file 3. [file elife-71035-supp3.docx]

**Supplementary Table 3.**

|  | **Histologies of model** | | | | | | | |  |
| --- | --- | --- | --- | --- | --- | --- | --- | --- | --- |
|  | ***All (including others)*** | |  | ***NSCLC*** | |  | ***SCLC*** | |  |
| **Time windows:** | **AUC** | **Av. # of features*** | **Av. % of acc/sn/sp** | **AUC** | **Av. # of features** | **Av. % acc/sn/sp** | **AUC** | **Av. # of features** | **Av. % acc/sn/sp** |
| 0-2 years | 0.72 (95% CI, 0.63-0.81) | 726 | 72/66/79 | 0.73 (95% CI, 0.66-0.81) | 37 | 75/80/70 | 0.64 (95% CI, 0.56-0.74) | 15 | 68/65/70 |
| 0-5 years | 0.74 (95% CI, 0.68-0.79) | 335 | 71/73/68 | 0.73 (95% CI 0.7-0.76) | 1362 | 71/80/62 | 0.70 (95% CI, 0.61-0.78) | 36 | 74/67/80 |
| 0-8 years | 0.74 (95% CI, 0.7-0.78) | 382 | 70/71/68 | 0.71 (95% CI, 0.65-0.78) | 201 | 68/70/66 | 0.76 (95% CI, 0.72-0.80) | 60 | 71/70/74 |
