# Supplementary material for: Serum RNAs can predict lung cancer up to 10 years prior to diagnosis
Source: eLife. 2022 Feb 11;11:e71035. doi: 10.7554/eLife.71035 (PMC8884722; doi:10.7554/eLife.71035)
Supplement: Supplementary file 4. [file elife-71035-supp4.docx]

**Supplementary Table 4.** Selected prior to diagnosis models

|  | **Prior to diagnosis Models** | | | |
| --- | --- | --- | --- | --- |
|  | **NSCLC (0-2)**** | **NSCLC (6-8)**** | **SCLC (2-5)**** | **SCLC (8-10)**** |
| **Features** | tRF-20-739P8WQ0  tRF-20-J4S2I7L7  RNU2-19P  hsa-miR-193a-5p  NUDT3  iso-21-B0NKZ0RJ0  RBM39  tRF-29-7EMQ18Y3E7IN  iso-23-B0NKZ01J0E  iso-22-80FOUHBBP  piR-hsa-26131  iso-21-DIPPZBOI0  iso-20-RNUW92OI  TANC1  iso-17-BJ93X24  iso-17-DIRN504  tRF-29-3IRW18V6XOIE  RP11-182L21.6  tRF-28-6SXMSL73VLD5  hsa-miR-375-3p  hsa-miR-184  tRF-35-I3Z9HMI8W47W1R | hsa-miR-1273h-5p  piR-hsa-27124  PTCH2  RN7SL40P  RN7SL617P  RNU2-20P  RNY4P16  RNY4P28  RNY4P9  iso-23-8K4P8R8SDE  tRF-9MV47P594  tRF-YP9LON4V3  tRF-KY7343RXI7  tRF-PSQP4PW3FJI0V | iso-23-B0NKZ01JDW  ATL3  iso-21-Q85XJJ70D  HMGB1  hsa-miR-19b-3p  hsa-miR-215-5p  hsa-miR-30a-5p  hsa-miR-339-3p  hsa-miR-760  RN7SL277P  TNFRSF13C | MARCH8  tRF-25-JY7383RPD9  tRF-27-Q1Q89P9L842  tRF-22-947673FE5  EXOC3  PTCH2  piR-hsa-1593  piR-hsa-28391  GRAP2  B4GALT2  ATP8A1  C6orf223  FADS1  iso-22-B04KZ01JL  MSN |
| **Total features** | 22 | 14 | 11 | 15 |
| **AUC** | 0.89 (95% CI, 0.84-0.96) | 0.82 (95% CI, 0.76-0.88) | 0.89 (95% CI, 0.77-1.0) | 0.83 (95%, 0.69-0.97) |
| **Mean Sensitivity** | 90% | 89% | 88% | 91% |
| **Mean Specificity** | 80% | 70% | 86% | 77% |
| **Mean Accuracy** | 85% | 80% | 87% | 84% |
