# Supplementary material for: Serum RNAs can predict lung cancer up to 10 years prior to diagnosis
Source: eLife. 2022 Feb 11;11:e71035. doi: 10.7554/eLife.71035 (PMC8884722; doi:10.7554/eLife.71035)
Supplement: Transparent reporting form [file elife-71035-transrepform1.docx]

***eLife’s* transparent reporting form**

We encourage authors to provide detailed information *within their submission* to facilitate the interpretation and replication of experiments. Authors can upload supporting documentation to indicate the use of appropriate reporting guidelines for health-related research (see [EQUATOR Network](http://www.equator-network.org/%20)), life science research (see the [BioSharing Information Resource](https://biosharing.org/)), or the [ARRIVE guidelines](http://www.plosbiology.org/article/info:doi/10.1371/journal.pbio.1000412) for reporting work involving animal research. Where applicable, authors should refer to any relevant reporting standards documents in this form.

If you have any questions, please consult our Journal Policies and/or contact us: [editorial@elifesciences.org](mailto:editorial@elifesciences.org).

**Sample-size estimation**

- You should state whether an appropriate sample size was computed when the study was being designed
- You should state the statistical method of sample size computation and any required assumptions
- If no explicit power analysis was used, you should describe how you decided what sample (replicate) size (number) to use

Please outline where this information can be found within the submission (e.g., sections or figure legends), or explain why this information doesn’t apply to your submission:

We did not perform an explicit power analysis since variance differs widely between RNAs. Cases and controls were selected from Janus Serum Bank Cohort containing prediagnostic serum samples, collected up to ten years before the lung cancer diagnosis (for case samples). Only individuals with smoking history (smokers) were included in this study as stated explicitly in materials and methods. Table 1 shows clinical and histological characteristics of samples.

**Replicates**

- You should report how often each experiment was performed
- You should include a definition of biological versus technical replication
- The data obtained should be provided and sufficient information should be provided to indicate the number of independent biological and/or technical replicates
- If you encountered any outliers, you should describe how these were handled
- Criteria for exclusion/inclusion of data should be clearly stated
- High-throughput sequence data should be uploaded before submission, with a private link for reviewers provided (these are available from both GEO and ArrayExpress)

Please outline where this information can be found within the submission (e.g., sections or figure legends), or explain why this information doesn’t apply to your submission:

The selection and exclusion criteria were explained in the manuscript (see materials and methods). We have assessed technical replicates and variance for control samples in a previous manuscript (Umu et al, RNA Biology, 2017). We have also published a Data Resource Profile study to explain the extent of our RNA-seq dataset including lung cancer ([www.medrxiv.org/content/10.1101/2021.01.22.20243154v1](http://www.medrxiv.org/content/10.1101/2021.01.22.20243154v1)) Our analyses includes only biological replicates in this study. The consort diagram (Figure 1A) shows how we performed sample selection/exclusion. Unfortunately, the datasets generated for this article are not readily available because of the principles and conditions set out in articles 6 (1) (e) and 9 (2) (j) of the General Data Protection Regulation (GDPR). National legal basis as per the Regulations on population-based health surveys and ethical approval from the Norwegian Regional Committee for

Medical and Health Research Ethics (REC) is also required. Requests to access the datasets should be directed to the corresponding authors with a research proposal. Commercial applications can also be directed. Please go to our project web page for the latest information: https://www.kreftregisteret.no/en/janusrna

**Statistical reporting**

- Statistical analysis methods should be described and justified
- Raw data should be presented in figures whenever informative to do so (typically when N per group is less than 10)
- For each experiment, you should identify the statistical tests used, exact values of N, definitions of center, methods of multiple test correction, and dispersion and precision measures (e.g., mean, median, SD, SEM, confidence intervals; and, for the major substantive results, a measure of effect size (e.g., Pearson's r, Cohen's d)
- Report exact p-values wherever possible alongside the summary statistics and 95% confidence intervals. These should be reported for all key questions and not only when the p-value is less than 0.05.3

Please outline where this information can be found within the submission (e.g., sections or figure legends), or explain why this information doesn’t apply to your submission:

In this study, we used machine learning classifiers. We defined the algorithms, packages and versions. We also compared algorithms and justified why we chose these (see materials and methods/ results section 1). Since we cannot share the RAW data, we shared the plotting data. Materials and methods section describes how we calculated confidence intervals. P value was only used once for the pathway analysis. We explained how we calculated this p value (see materials and methods) and how we adjusted for multiple testing.

(For large datasets, or papers with a very large number of statistical tests, you may upload a single table file with tests, Ns, etc., with reference to sections in the manuscript.)

**Group allocation**

- Indicate how samples were allocated into experimental groups (in the case of clinical studies, please specify allocation to treatment method); if randomization was used, please also state if restricted randomization was applied
- Indicate if masking was used during group allocation, data collection and/or data analysis

Please outline where this information can be found within the submission (e.g., sections or figure legends), or explain why this information doesn’t apply to your submission:

NA

**Additional data files (“source data”)**

- We encourage you to upload relevant additional data files, such as numerical data that are represented as a graph in a figure, or as a summary table
- Where provided, these should be in the most useful format, and they can be uploaded as “Source data” files linked to a main figure or table
- Include model definition files including the full list of parameters used
- Include code used for data analysis (e.g., R, MatLab)
- Avoid stating that data files are “available upon request”

Please indicate the figures or tables for which source data files have been provided:

We shared all our analyses/modelling functions and bioinformatics workflow files on Github (<https://github.com/sinanugur/LCscripts>). We also shared tables that were used to create the plots. Any parameters of functions are also accessible from the Github repo. We included supplementary tables 1 and 2 to our submission. These tables contain the selected features and feature importance values of models.
